# Health Literacy, Social Determinants of Health, and Disease Prevention and Control

**Published:** 2020-12-16

**Authors:** Steven S. Coughlin, Marlo Vernon, Christos Hatzigeorgiou, Varghese George

**Affiliations:** 1 Department of Population Health Sciences, Medical College of Georgia, Augusta University, Augusta, GA; 2 Institute of Public and Preventive Health, Augusta University, Augusta, GA; 3 Georgia Cancer Center, Augusta University, Augusta, GA; 4 Department of Medicine, Medical College of Georgia, Augusta University, Augusta, GA

## Introduction

Health literacy has been defined as the “degree to which individuals have the capacity to obtain, process and understand basic health information and services needed to make appropriate health decisions” (Nielsen-Bohlman et al., 2004). Low health literacy is associated with more hospitalizations, greater use of emergency care, decreased use of preventive services, poorer ability to interpret labels and health messages, poorer health status, higher mortality, and higher health care costs (Berkman et al., 2011). Functional health literacy extends beyond proficiency in reading, writing, and numeracy to include interpretation of images and oral communication (Magnani et al., 2018; Ousseine 2019). Communicative health literacy is essential to abstract skills such as evaluating and weighing treatment considerations and engaging in medical decision-making (Magnani et al., 2018; Ousseine 2019). Low health literacy negatively impacts disease self-management and individual health behaviors such as adherence with weight control and tobacco cessation interventions and cancer screening recommendations (Weiss & Smith-Simone, 2010; Bennett et al., 2009). Individuals with low health literacy are more likely to present with advanced illness, resulting in delayed diagnosis and treatment and poorer outcomes (Aljassim & Ostini, 2020).

Health literacy is recognized not only as an individual trait or risk factor for poorer health outcomes, but also as an asset or characteristic related to families, communities, and organizations that provide health and social services (Batterham et al., 2016). Viewed as an asset, health literacy offers a means to empower individuals and communities to exert greater control over their health (Aljassim & Ostini, 2020; Nutbeam, 2008).

Nevertheless, many individuals are unable to comprehend or act upon health information because of limited health literacy (Fleary & Ettienne, 2019). The National Assessment of Adult Literacy Survey found that 36% of U.S. adults had basic or below-basic health literacy (Magnani et al., 2018). In the U.S., non-whites are more likely to have limited health literacy than whites (Mantwill et al., 2015; Berkman et al. 2011; Kutner et al., 2006). Low socioeconomic status, particularly low educational attainment, is the most important determinant of health literacy (Stormacq et al., 2019; Garcia-Cordina et al. 2019). Lower health literacy is associated with income and education (von Wagner et al., 2007; Paasche-Orlow et al., 2005). Rates of limited health literacy are also higher among elderly persons and among non-native English speakers (Berkman et al., 2011; Fleary & Ettienne, 2019).

Shared decision making (SDM) is often heralded as a key component of patient-centered health care, but effective patient and provider communication relies on a degree of health literacy. Patients must be able to understand and process medical information in order to make informed decisions about their health care. Interventions which incorporate simple design, plain language, and graphic displays have made improvements in SDM in disadvantaged populations to a greater degree than those with higher literacy (Durand 2014). Other research proposes that information which is appropriately designed and culturally competent could contribute to reductions in cancer and other health disparities (Polaceck 2007, Hawley 2017).

Health literacy is an important factor in disease prevention and control (Amalraj et al., 2009; Davis et al., 2002; Halverson et al., 2013). Adults with limited health literacy obtain less information from disease prevention and control materials, and may be less likely to undergo screening or to successfully manage their illness. For example, health literacy is associated with obesity, dietary choices, and exercise (Magnani et al., 2018). Women with low health literacy are also associated with a lower probability of mammography screening; they are also more likely to report poorer physician-patient communication and higher levels of decision regret in regard to their breast cancer decisions (Berkman et al., 2011; Durand 2020). Low health literacy is also associated with having an inadequate understanding of complex medical information (Davis et al., 2002; Halverson et al., 2013). Patients’ understanding of their disease, treatment, and healthcare decision-making can be impaired by low health literacy (Halverson et al., 2013).

Health literacy is associated with other determinants of health (e.g., education, income, area-based measures of social disadvantage, and access to healthcare) that are key to the success of disease prevention and control efforts aimed at health disparities (Simmons et al., 2017). Interventions that improve health literacy may empower individuals and communities to take action on social and economic determinants of health at both the individual and community level. Improvements in health literacy are likely to result in improved utilization of preventive services, medical adherence, and involvement in health decision-making (Fleary & Ettienne, 2018). Effective interventions to improve health literacy may include interventions to improve patient-provider communication or to develop skills in low literate people (Rowlands et al., 2017). Growing literature in the developing field of health literacy continues to show that interventions do make a difference and can positively impact behaviors that ultimately decrease disease burden (Miller, 2016). If more progress and closer links are established with health literacy and behavior change theories and theoretical frameworks, additional efficacy is possible (Walters et al., 2020). Thus, health literacy is a potentially modifiable factor by which disparities in disease morbidity and mortality can be reduced (Mantwill et al. 2015). Enhancing the level of health literacy in the population or making health services more accessible to people with low health literacy may offer the means to achieve greater equity in disease outcomes (Stormacq et al., 2018).
